# A system for site-specific integration of transgenes in mammalian cells

**DOI:** 10.1371/journal.pone.0219842

**Published:** 2019-07-25

**Authors:** Xiuling Chi, Qi Zheng, Ruhong Jiang, Ruby Yanru Chen-Tsai, Ling-Jie Kong

**Affiliations:** Applied StemCell, Inc., Milpitas, California, United States of America; Chinese Academy of Sciences, CHINA

## Abstract

Mammalian cell expression systems are the most commonly used platforms for producing biotherapeutic proteins. However, development of recombinant mammalian cell lines is often hindered by the unstable and variable transgene expression associated with random integration. We have developed an efficient strategy for site-specific integration of genes of interest (GOIs). This method enables rapid and precise insertion of a gene expression cassette at defined loci in mammalian cells, resulting in homogeneous transgene expression. We identified the Hipp11 (H11) gene as a "safe harbor" locus for gene knock-in in CHO-S cells. Using clustered regularly interspaced short palindromic repeats (CRISPR)/Cas9 mediated homologous recombination, we knocked in a DNA cassette (the landing pad) that includes a pair of PhiC31 bacteriophage attP sites and genes facilitating integrase-based GOI integration. A master cell line, with the landing pad inserted correctly in the H11 locus, was established. This master cell line was used for site-specific, irreversible recombination, catalyzed by PhiC31 integrase. Using this system, an integration efficiency of 97.7% was achieved with green fluorescent protein (GFP) after selection. The system was then further validated in HEK293T cells, using an analogous protocol to insert the GFP gene at the ROSA26 locus, resulting in 90.7% GFP-positive cells after selection. In comparison, random insertion yielded 0.68% and 1.32% GFP-positive cells in the CHO-S and HEK293T cells, respectively. Taken together, these findings demonstrated an accurate and effective protocol for generating recombinant cell lines to provide consistent protein production. Its likely broad applicability was illustrated here in two cell lines, CHO-S and HEK293T, using two different genomic loci as integration sites. Thus, the system is potentially valuable for biomanufacturing therapeutic proteins.

## Introduction

Over several decades, the Chinese hamster ovary (CHO) cell has served as the major standard expression platform for producing recombinant therapeutic proteins [1, 2]. The traditional approach for developing recombinant CHO cell lines is random insertion of the gene of interest into the genome, followed by a selection for cells carrying the transgene [1, 2]. However, unstable, variable transgene expression has been a primary limitation, decreasing the consistency and homogeneity of cell-based protein production. With the CHO genomic sequence, it is now feasible to accurately engineer the host cell genome. Site-specific integration has been performed in CHO cell lines using a variety of technologies, including Cre-Lox [3, 4], Flp-FRT [5, 6], zinc finger nucleases (ZFNs) [7], transcription activator-like effector nucleases (TALENs) [8, 9] and CRISPR/Cas9 [10, 11]. With these technologies, integration efficiency and accuracy were enhanced compared with use of random knock-in methods. Moreover, site-specific integration can lead to homogeneous, stable gene expression at a predefined locus for consistent protein production. One of the site-specific integration strategies is to take the advantage of the unidirectional integration property of bacteriophage PhiC31 integrase. The PhiC31 integrase is a sequence-specific recombinase encoded within the genome of the bacteriophage PhiC31. The PhiC31 integrase mediates precise, unidirectional recombination between two att sites named attB and attP. This integrase has been shown to function efficiently in many different cell types as well as in mice [12, 13]. A major drawback of current methods for site-specific integration is that the procedures required to obtain the final single cell clone are lengthy and laborious. In addition, during the process of integration, the construct backbone may remain incorporated into the genome, resulting in such side effects as gene silencing. Of further concern, the genetic and epigenetic properties of a cell line derived from a single targeted-integration clone may change over time, even when cells are maintained under constant conditions [14, 15]. Here, we developed a site-specific integration system based on PhiC31 integrase, termed "TARGATT", coupled with short-term negative selection, for precise gene insertion in the CHO-S genome and generation of a stable cell line. This system eliminated some of the drawbacks of random integration, and serves as a fast and efficient integration platform for generating a uniform cell population with stable transgene expression.

## Materials and methods

### Vector construction

The gRNA design and cloning were performed using Cas9 plasmid pX458 as previously described [16]. The targeting sequences for the CHO-S Hipp11 site were cH11g1: 5’- GTATACACTTGAGCCAGTAGTGG -3’ and cH11g2: 5’- TATACACTTGAGCCAGTAGTGGG -3’. The targeting sequence for the human ROSA26 site was 5’-AGTCGCTTCTCGATTATGGGCGG-3’. The TARGATT landing pad was synthesized by Genscript (Piscataway, NJ, USA). At each end of the cassette was a PhiC31 bacteriophage attP site. To construct the CRISPR donor for the CHO-S Hipp11 site, genomic DNA from CHO-S cells was used as template for PCR of the 5’ homologous arm, with primer set 5’-TTGCATATGCCACGTGTGTGAAC-3’ and 5’-ACTGGCTCAAGTGTATACTTGGG-3’, and the 3’ homologous arm, with primer set 5’-AAGAATTACTGTGCAGGCTGAAG-3’ and 5’-TATGTCTATGCACTATTTGTGTC-3’. The landing pad was ligated to the homologous arms and cloned into pUC19. A similar strategy was used for the CRISPR donor plasmid for human ROSA26, using human genomic DNA from HEK293T cells and a human ROSA26 PCR primer set. The primers for the 5’ homologous arm were 5’-GGTTTGTTGGACTTAGCTTTCAGC-3’ and 5’- GGGCGGGATTCTTTTGCCTAGGC-3’ and those for the 3’ homologous arm were 5’- ATAATCGAGAAGCGACTCGACAT-3’ and 5’-TGAGGTTCGGTCTCTTTTCTGTC-3’. The TARGATT GFP donor for integrase dependent targeting contained the GFP gene under a CAG promoter and an SV40 PolyA. The GFP expression cassette had a PhiC31 attB site at each end. Another HSV-TK expression cassette with a PGK promoter and SV40 PolyA was cloned downstream of the 3’ attB site. The two cassettes were synthesized by Genscript and cloned into a pUC19 plasmid. The size of the donor vector is 8013bp.

### Cell culture

CHO-S cells were maintained in suspension in BalanCD CHO growth A medium (Irvine Scientific, Santa Ana, CA, USA) supplemented with 6 mM Gibco GlutaMAX (Thermo Fisher, Waltham, MA, USA) and 100 μg/ml penicillin and streptomycin. HEK293T cells were maintained in Dulbecco's modified Eagle's Medium (DMEM) high glucose with 1 mM sodium pyruvate, 2 mM L-glutamine and 10% fetal bovine serum. Both cell lines were cultured at 37°C in a humidified incubator equilibrated with 95% air, 5% CO_2_.

### gRNA validation

To validate CHO-S cell gRNA, individual gRNA constructs were transfected into CHO-S cells with a single 1500 V pulse for 30 ms using a Neon electroporator (Invitrogen, Carlsbad, CA, USA). Cells were cultured for 5 days. DNA was then extracted and used as a template for PCR with the primer set 5’-TGGATTTTGACTGCAGGGGTAAA-3’ and 5’-CTGTGGCTTTGGAGCCTACACTG-3’. A 674bp PCR fragment was amplified. To determine gRNA activity, a surveyor assay was performed on the PCR product, using the T7 endonuclease I-based mutation detection Kit (New England Biolabs Inc., Ipswich, MA, USA), according to the manufacturer’s instructions. To validate human gRNA, the protocol was analogous, but using HEK293T cells and lipofectamine 2000 (Invitrogen, Carlsbad, CA) instead of electroporation for transfection.

### Transfections

Approximately 1 X 10^6^ CHO-S cells were transfected with 2 μg plasmid DNA using a single 1500 V, 30 ms electroporation pulse. Cells were then transferred to a six-well plate containing culture medium. Cell viabilities and densities were monitored with an automated cell viability analyzer, the Countess II FL automated cell counter (Invitrogen, Carlsbad, CA). Medium was replenished every two to three days. For HEK293T cells, lipofectamine 2000 was used for transfection. For puromycin selection, puromycin was added to the medium three days after transfection and cells were cultured for seven days. The puromycin concentration used for CHO-S and HEK293T cells was 8 and 1 μg/ml, respectively. For TARGATT integration selection, 1 μg/ml GCV was added to cells on day seven after transfection and cells were incubated for additional three days. GCV was then removed and cells were maintained in normal culture medium without GCV.

### Fluorescence microscopy

Approximately three weeks after transfection of the GFP donor plasmid into the master cell lines, images from cells treated with or without GCV were obtained with a Leica DFC 500 fluorescence microscope, using the bright-field and green fluorescence channels. All images were acquired under magnification with a 20x objective.

### PCR

Genomic DNA from CHO-S and HEK293T cell lines was isolated using QuickExtract DNA Extraction Solution (Epicentre, Madison, WI, USA), and was then used as a template for PCR amplification. PCR was performed using the GoTaq Green Master Mix kit from Promega (Madison, WI, USA). Reaction conditions were: 95°C for 2 min; 35 cycles of 95°C for 30 s, 58°C for 30 s, 72°C for 2 min; 72°C for 10 min. Primers used for genotyping of the CHO-S master cell line were: 5’-arm-Forward: 5’-TGGAGCCTACACGGTCCAAAT-3’; 5’-arm-Reverse: 5’-CCTACCCGCTTCCATTGCTCAG-3’; 3’-arm-Forward: 5’-CACCTCCCCCTGAACCTGAAAC-3’; 3’-arm-Reverse: 5’-CCCTCGAAAGGACTTCTTGCTC-3’. Primers used for genotyping of the CHO-S stable cell line at the 5’- and 3’-arms were: 5’-arm-Forward: 5’-ACCTGGAAAAAGCAGTCCCAAC-3’; 5’-arm-Reverse: 5’-ACACCTCCCCCTGAACCTGAA-3’; 3’-arm-Forward: 5’-GGCGGGCCATTTACCGTAAGTTAT-3’; 3’-arm-Reverse: 5’-CTACCGGTGGATGTGGAATGTGT-3’. Primers used for genotyping of the HEK293T master cell line were: 5’-arm-Forward: 5’-CTCCAGACTGCCTTGGGAAAAGC-3’; 5’-arm-Reverse: 5’-GTAGCTCCTGTAACAGTTTAATGGAATCTCACC-3’; 3’-arm-Forward: 5’-GGTGAGAAGTGGCAGCATCCT-3’; 3’-arm-Reverse: 5’-CTAGATAACTGATCATAATCAGCCATACCACAT-3’. Primers used for genotyping of the HEK293T stable cell line at the 5’- and 3’-arms were: 5’-arm-Forward: 5’-ATCCGCAGTCTCGTTGCATAAAATCAG-3’; 5’-arm-Reverse: 5’-CATCCTGGTCGAGCTGGACG-3’; 3’-arm-Forward: 5’-CTTTATGGGAGTTCTCTGCTGCCTC-3’; 3’-arm-Reverse: 5’-TCAATGGGCGGGGGTCGTTG-3’.

### Fluorescence-activated cell sorting (FACS)

Cells were analyzed for GFP expression, before and after selection, using a BD FACS Melody (San Jose, CA, USA) with a 488 nm laser. CHO-S and HEK293T parental cell lines were used to determine the gating for GFP-positive cells. After delivery of targeting vectors, the percentages of cells expressing GFP, with or without GCV selection, were analyzed for each cell type under each condition.

### Western blotting

The cell samples from CHO-S and HEK293T cells were harvested and washed twice with PBS and lysed with RIPA buffer (Pierce, Appleton, WI). The concentration of lysate was measured using the BCA assay (Pierce, Appleton, WI). Equal amounts of lysate protein were run in a 10% Bis-Tris NUPAGE gel (Invitrogen, Carlsbad, CA) and were then transferred to a PVDF membrane using an iBlot2 machine (Invitrogen, Carlsbad, CA). Immunoblots were incubated using an anti-GFP rabbit primary antibody (Abcam, Cambridge, MA) and anti-rabbit IgG-HRP secondary antibody (Abcam, Cambridge, MA). Blots were incubated with Supersignal extended substrate (Thermo Fisher, Waltham, MA) and then imaged with an iBright CL1000 system (Invitrogen). Anti-alpha-tubulin antibody (Abcam, Cambridge, MA) was used as the loading control for both cell lines.

### Determination of gene copy numbers

Copy numbers of GFP from CHO-S and HEK293T cells after GCV selection were quantified via Droplet Digital PCR (ddPCR) using the QX200 system (Bio-Rad Laboratories, Hercules, CA). Genomic DNA was extracted and then digested with EcoRI before the ddPCR reactions. Each sample was run in duplicate. Two sets of primer/probe, one specific for GFP, and the other specific for an endogenous control, were used to determine the copy number for each target gene. The genes Cog1 and C1orf159 were used as endogenous controls for CHO-S and HEK293T cells, respectively. Primers and probes were synthesized by Integrated DNA Technologies (San Diego, CA). The primers used for detection of GFP in CHO-S and HEK293T cells were: Forward: 5’- GACTTCTTCAAGTCCGCCAT-3’; Reverse: 5’-CAACTACAACAGCCACAACG-3’. The probe used to detect GFP was: 5’-CGACGGCAACTACAAGACCC-3’-FAM. The primers used to detect Cog1 in CHO-S cells were: Forward: 5’-ACCGTTAAGAACACACAGCA-3’; Reverse: 5’- ATGGCGTCCTACCAAAACAA-3’; The probe used to detect Cog-1 in CHO-S cells was: 5’-CCCTTTGTCTTGCGGATGGT-3’-HEX. The primers used to detect the C1orf159 gene in HEK293T cells were: Forward: 5’-CAGCAATGTCTGACCTGGAG-3’; Reverse: 5’-CCGATAAACGAGATGGCTGT-3’. The probe used to detect C1orf159 in HEK293T cells was: 5’-CACAAAGTGTTGGCATCGCC-3’-HEX;

### Off-target analysis of CRISPR/Cas9

Using the sequence of cH11g2 for the CRISPR/Cas9 system and all four possible PAM sequences NGG (AGG, TGG, CGG and GGG) at the 3′ end, a BLAST of the CHO genome was performed to determine the top 5 sites that are most similar to cH11g2. Five sets of primers were designed to amplify these potential off-target sites. Primers used for amplifying the 5 potential off-target sites were: Site 1: Forward: 5’-CACTGAAGTCATTTCACCACCTCA-3’; Reverse: 5’-AGAGAGGACGTGCTGGCTTTTTA-3’; Site 2: Forward: 5’-GGGGAGGATAGGTGTGAACAGAA-3’; Reverse: 5’-GGGTCCCTTCCTATGAAAAGCTG-3’; Site 3: Forward: 5’- TGGGGTGAAGTCTATACCCAGTGA-3’; Reverse: 5’-GACATCAGAAAGGGGAAGCAAGA-3’; Site 4: Forward: 5’-TGTAATGTCAACATGCGCCTACC-3’; Reverse: 5’-CGGGCCTTCTTGTATCAGTCAGT-3’; Site 5: Forward: 5’-CAGCAACCAGAGAAGGAAAGGAA-3’; Reverse: 5’-CCCCTGCCCAGCAATACTAATAA-3’. Sanger sequencing was performed on the PCR products to detect any off-target events.

## Results

### Generation of a CHO-S master cell line at the H11 locus

#### Identification of the H11 locus as the integration site

A "safe harbor" genomic locus is crucial for efficient transgene knock-in and expression. The Hipp11 (H11) locus, which is situated between the DRG1 and EIF4ENIF1 genes in mice, humans, and pigs, offers great potential for stable gene knock-in and expression at high levels [17, 18]. The robust and ubiquitous function of H11 has been demonstrated in mice, pigs, human embryonic stem (hES) cells, and induced pluripotent stem (iPS) cells [17–19].

**Fig 1:**
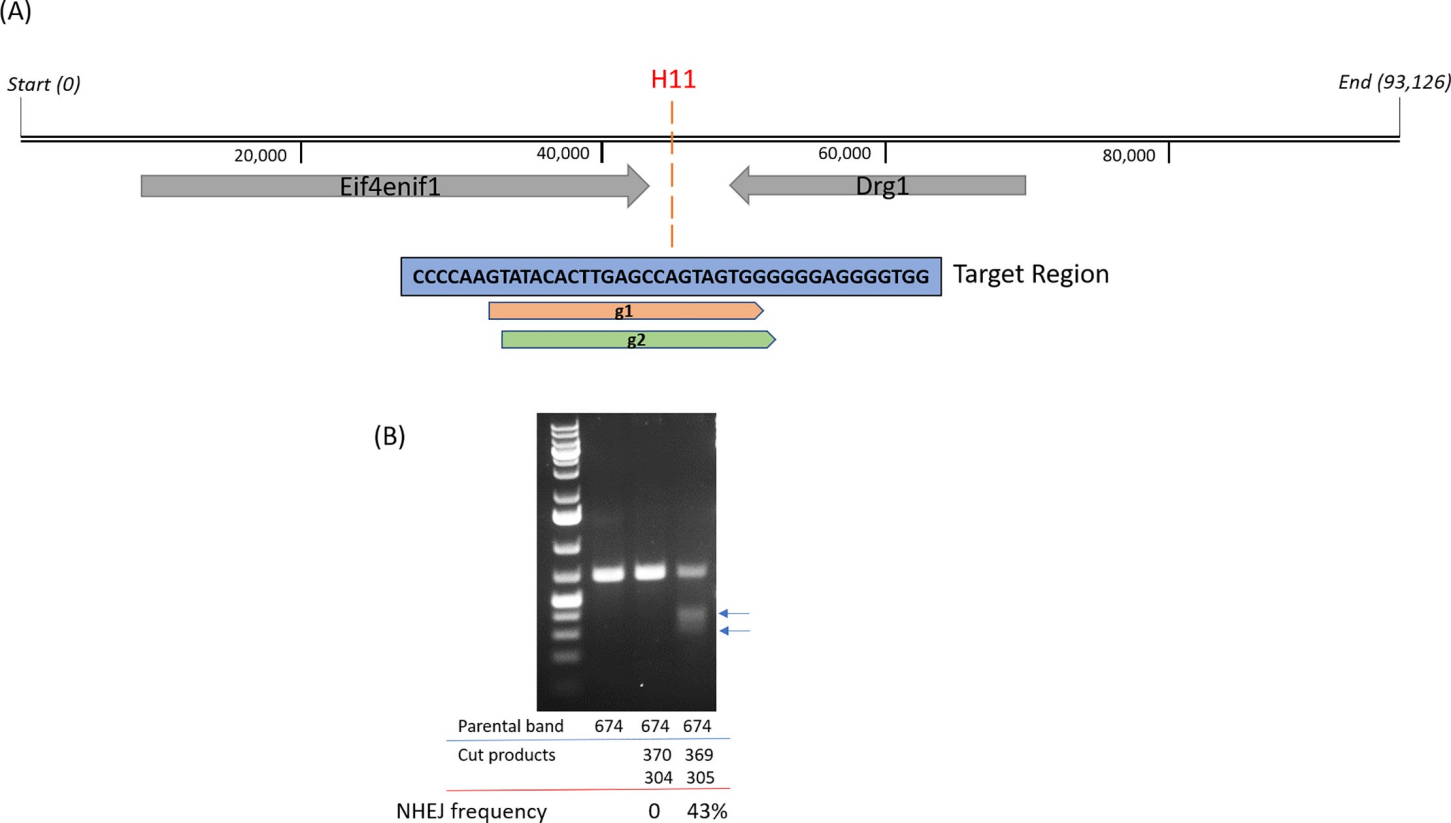
Map of the H11 locus in the CHO-S genome and gRNA validation. (A) The genomic region between the EIF4ENIF1 and DRG1 genes was targeted for CRISPR knock-in. Targeting locations of the two gRNA candidates, g1 and g2, are shown. (B) For gRNA validation, CHO-S cells were electroporated with the indicated Cas9–gRNA constructs and gRNA activity evaluated by the surveyor assay. Lane 1, DNA marker; Lane 2, cells transfected with Cas9 plasmid only; Lane 3, cells transfected with Cas9–g1; Lane 4, cells transfected with Cas9–g2.

Public availability of the full genomic sequence of CHO-S cells [20] made it possible for us to identify the orthologous H11 locus via standard bioinformatics. Based on previously reported information about the H11 locus, we chose the location between the DRG1 and EIF4ENIF1 genes in the CHO-S cell genome for our study (Fig 1A). Based on data from transgenic mice, human stem cells and pigs [17–19], we hypothesized that this region would serve as a "safe harbor" to provide robust and active transgene expression in CHO-S cells.

#### CHO-S master cell line generation

**Fig 2:**
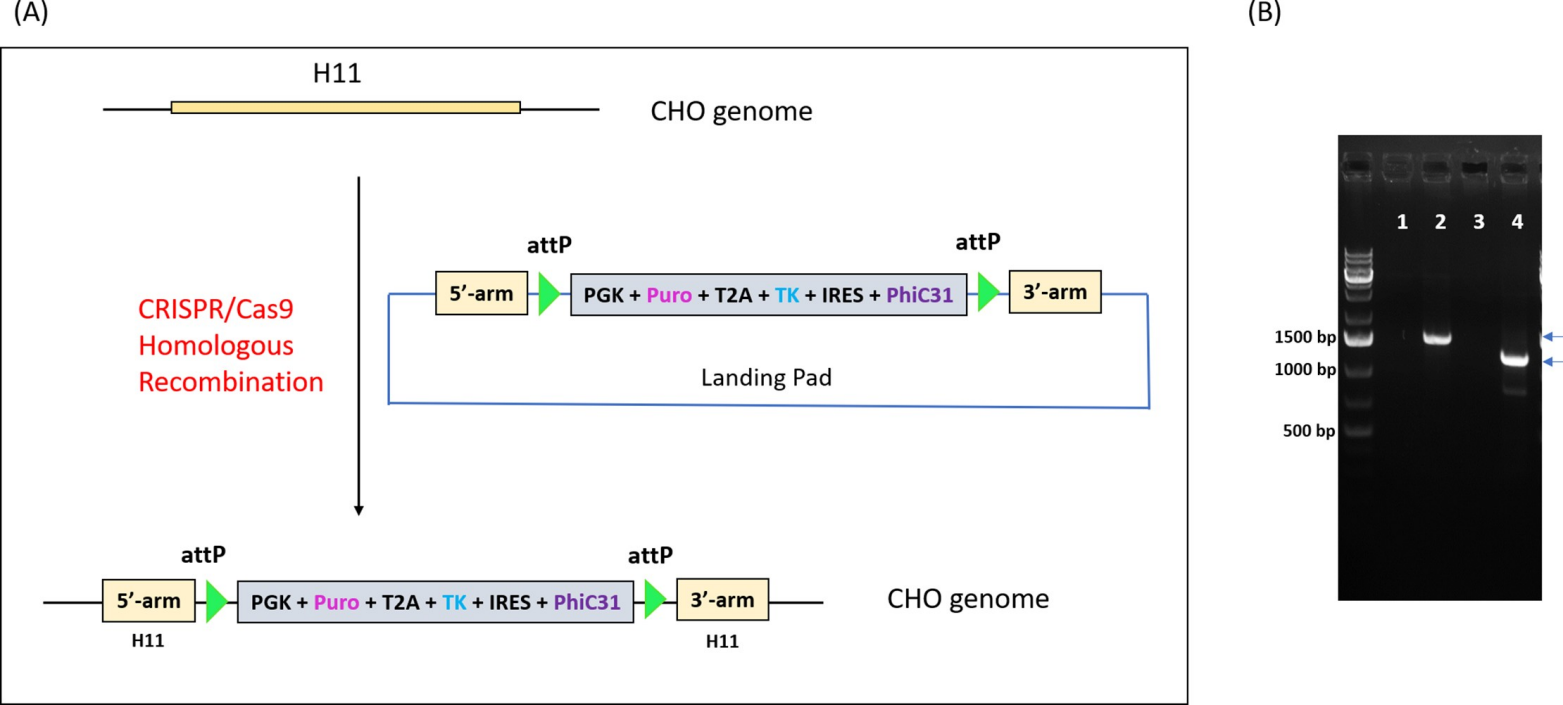
CHO-S master cell line generation. (A) The H11 locus was cleaved, using CRISPR/Cas9, to encourage integration of the landing pad donor. A successful knock-in at the H11 locus generates a master cell line that has two PhiC31 attP sites, and that co-expresses three proteins via a PGK promoter-driven transcript. (B) Genotyping of the 5’-arm and 3’-arm in the CHO-S master cell line. PCR was performed with genomic DNA and specific primer pairs. 1: CHO-S parental cell line with 5’-arm primers; 2: CHO-S master cell line ("4–6") with 5’-arm primers; 3: CHO-S parental cell line with 3’-arm primers; 4: CHO-S master cell line ("4–6") with 3’-arm primers.

To make the CHO-S master cell line, CRISPR/Cas9-mediated homologous recombination was used to insert the landing pad into the H11 locus of CHO-S parental cells. Two guide RNA (gRNA) candidates were designed and tested for promoting Cas9 cleavage in the H11 target region (Fig 1A). Their activities were evaluated by the surveyor nuclease assay [21]. Results showed that the indel frequency was 0% and 43% for gRNA1 and gRNA2, respectively. This indicated that gRNA2 worked well, resulting in the expected cleavage products, while gRNA1 did not (Fig 1B). Therefore, gRNA2 was chosen for CRISPR/Cas9-mediated integration of the landing pad into the H11 locus in the CHO-S cell genome. As shown in Fig 2A, the landing pad donor has a cassette driven by the PGK promoter that co-expresses a puromycin resistance protein, Herpes Simplex Virus-1 thymidine kinase (HSV-TK), and PhiC31 integrase. This cassette is flanked by a pair of PhiC31 attP sites. To co-express these proteins without fusing them, we made use of a *thosea asigna virus 2A* (T2A) ribosome-skipping sequence and also an internal ribosome entry site (IRES).

For inserting the designed landing pad into the CHO-S parental cell line, cells were transfected with both the Cas9–gRNA2 plasmid and the landing pad donor. Three days after electroporation, puromycin selection was applied to the cultured cells for seven days. Cells were then subjected to single cell cloning. Genotyping was performed on the single cell clones to confirm correct insertion of the landing pad into the H11 locus (Fig 2B). For genotyping, two pairs of site-specific primers, covering the space between the sites of 5’-arm and attP and 3’-arm and attP, were separately designed. To confirm correct insertion, genomic DNA was extracted from both CHO-S parental and master cell lines and PCR was performed. A single cell clone, named "4–6", was identified. As shown in Fig 2B, the clone showed DNA bands of the correct sizes on an agarose gel. DNA sequencing results of the PCR products showed that the landing pad had been inserted as expected into the H11 locus (**S1 Fig**). Finally, this clone was propagated as the CHO-S master cell line and used for stable cell line generation by TARGATT recombination.

To evaluate the possible off-target effects of CRISPR, we did in silico analysis (http://crispor.tefor.net/) to identify potential off-target sites inside the CHO-S genome. The top 5 potential off-targets sites with either 3 or 4 base mismatches were identified (**S2**A & **S2**B **Fig**). Sequence analysis of the PCR products from these regions showed that no gene editing events happened in the master cell line (**S2**C **Fig**).

### TARGATT knock-in of a GOI into the CHO-S master cell line

**Fig 3:**
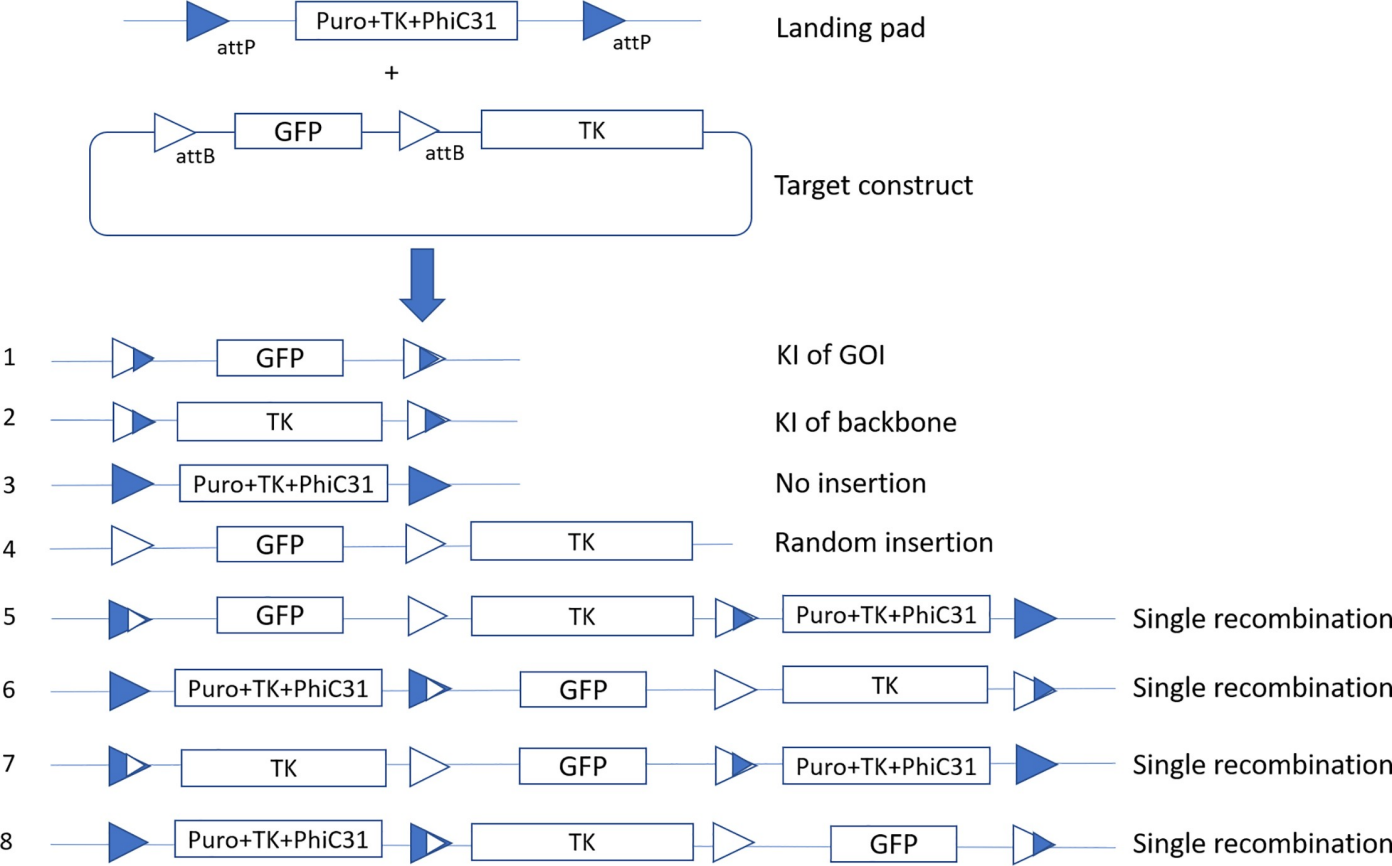
Schematic description of the TARGATT system. The two attP sites from the landing pad and the two attB sites from the donor construct are recombined to form eight possible recombination outcomes. The attP and attB sites highlighted in blue and white, respectively.

We constructed the TARGATT site-specific integration donor vector with the GOI flanked by a pair of attB sites. To demonstrate effectiveness of the system, GFP was used as a reporter gene. An HSV-TK gene was also introduced in the backbone of the donor vector, enabling negative selection with ganciclovir (GCV) to eliminate cells with unwanted recombination events (Fig 3**)**.

When the donor construct is transfected into the CHO-S master cell line, site-specific integration can be catalyzed by the PhiC31 integrase expressed in the cell. Recombination events between the donor and genome can occur between one or two attP–attB pairs, leading to total of eight possible recombination outcomes (Fig 3). All integration events between one attP–attB pair (Fig 3, (5–8)), will eventually lead to another recombination event because of the presence of integrase and a second pair of intact attP and attB sites. This will result in the same genotypes as those formed from recombination events involving both attP–attB pairs (Fig 3, (1–2)). In the end, there will be four types of cells in the transfected line, the ones with GFP only, backbone only, random integration, and no integration (Fig 3, (1–4)). When GCV is added, only the cells with on-target knock-in (Fig 3, (1)) will survive the selection, as they do not express HSV-TK. The cell line finally obtained will have neither the puro–HSV-TK–PhiC31 gene nor the plasmid backbone, only the GOI at the targeted location in the genome.

**Fig 4:**
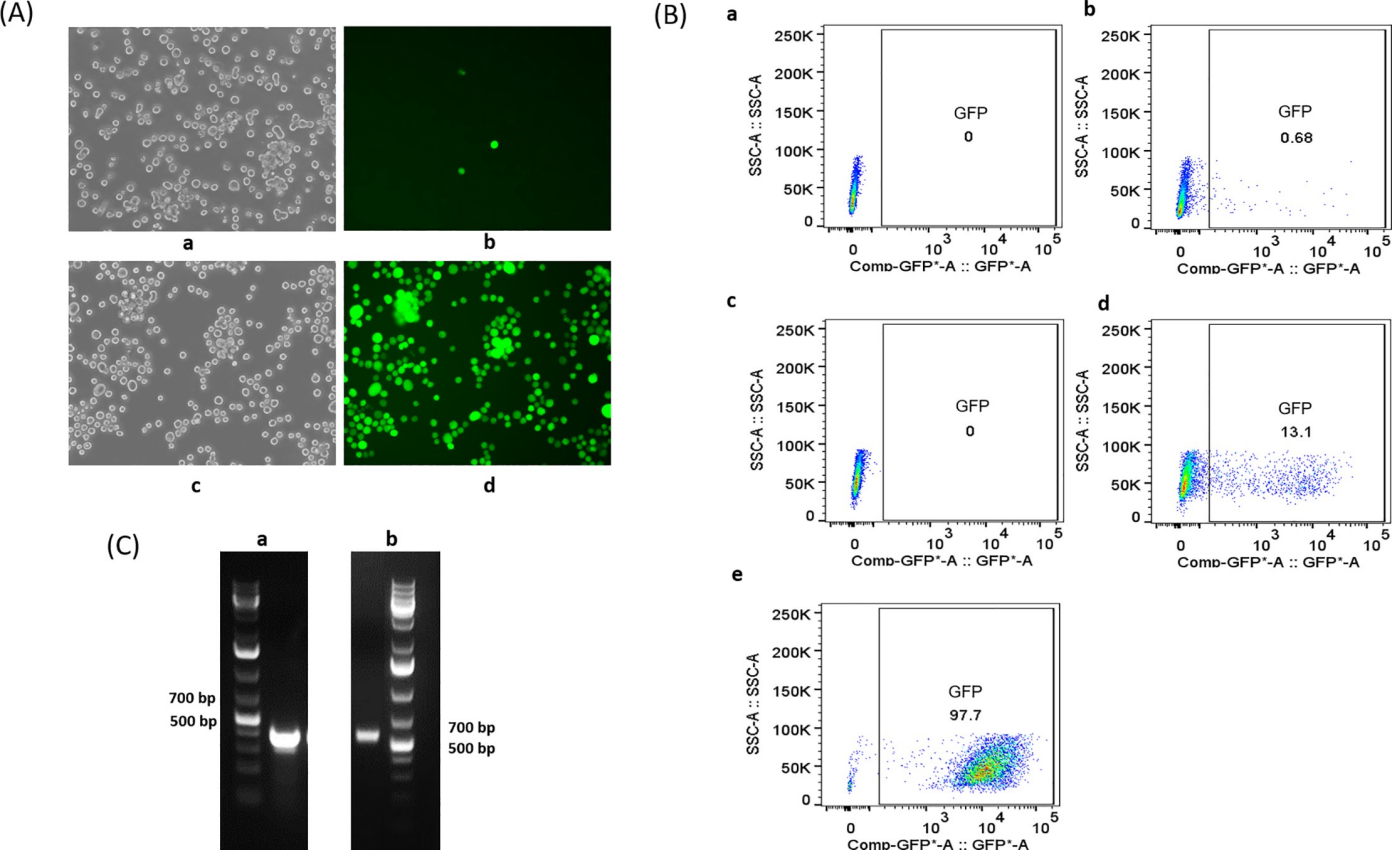
Evaluation of the efficiency of TARGATT site-specific integration in the CHO-S master cell line. (A) GFP signals were detected by fluorescence imaging in cells transfected with the donor construct: by random insertion, shown in bright-field (a) and GFP channel (b); and by TARGATT integration plus GCV selection in bright-field (c) and GFP channel (d). (B) FACS analysis of GFP expression in CHO-S cells. a, CHO-S parental cell line only; b, CHO-S parental cell line transfected with the donor plasmid; c, CHO-S master cell line only; d, CHO-S master cell line transfected with the TARGATT donor plasmid, prior to GCV selection; e, CHO-S master cell line transfected with the donor plasmid, after GCV selection. (C) Genotyping of the 5’-arm and 3’-arm junctions to confirm the correct recombination. PCR analysis showed the expected PCR product size, on a 1.5% agarose gel.

We transfected the donor construct into the CHO-S master cell line. Cells were cultured for two weeks to let the signal from transient transfection vanish. GFP expression was assessed by fluorescence microscopy and then analyzed by FACS. We used CHO-S parental cells lacking the attP docking sites under the same conditions as a control. Only random insertion events could happen in the control cells. As shown by fluorescence microscopy, almost all cells from the TARGATT master cell line treated with GCV were GFP-positive, while very few control cells were green (Fig 4A). FACS analysis showed that 13.1% of cells from the master cell line were GFP-positive prior to GCV selection, while only 0.68% of cells were GFP-positive from the control cell line (Fig 4B, 4D and 4B). This indicated that the TARGATT technology worked much more efficiently than random integration in CHO-S cells. After GCV selection, the percentage of GFP-positive cells derived from the transfected master cell line increased to 97.7% (Fig 4B and 4E). Furthermore, the FACS spectra indicated that GFP signal strength in TARGATT cells was more uniform and much more intense than in control cells (Fig 4B, 4E and 4B). The TARGATT system generated a cell line with a more uniform population in protein expression than random integration did. In addition, based on FACS analysis, GFP expression was maintained at stable levels after extensive passaging (>40 passages), supporting the potential for stable expression at the H11 locus in CHO-S cells. Western blotting was performed to detect the GFP protein expressed from CHO-S master cells that had been transfected with the GFP donor plasmid, both before and after GCV selection (S3 Fig, lanes 4–6). As expected, an increase in GFP protein was observed in the GCV-selected CHO-S cell line.

To validate on-target knock-in and correct recombination between the attP and attB docking sites in the CHO-S master cell line, we designed primers to detect the attP–attB recombination junctions and performed PCR. Gel analysis showed the expected PCR product sizes of 386 bp and 568 bp at the 5’-arm and 3’-arm, respectively (Fig 4C). DNA sequencing results of the PCR products showed the correct recombination sequences (S4 Fig). This confirmed that site-specific cassette exchange had occurred by TARGATT. Droplet digital PCR analysis showed we have a single copy of the GFP gene in the GCV-selected CHO-S cell pools (S5 Fig), confirming that our CHO master cell line can be used as a single-copy transgene integration system.

### TARGATT knock-in to HEK293T cells

**Fig 5:**
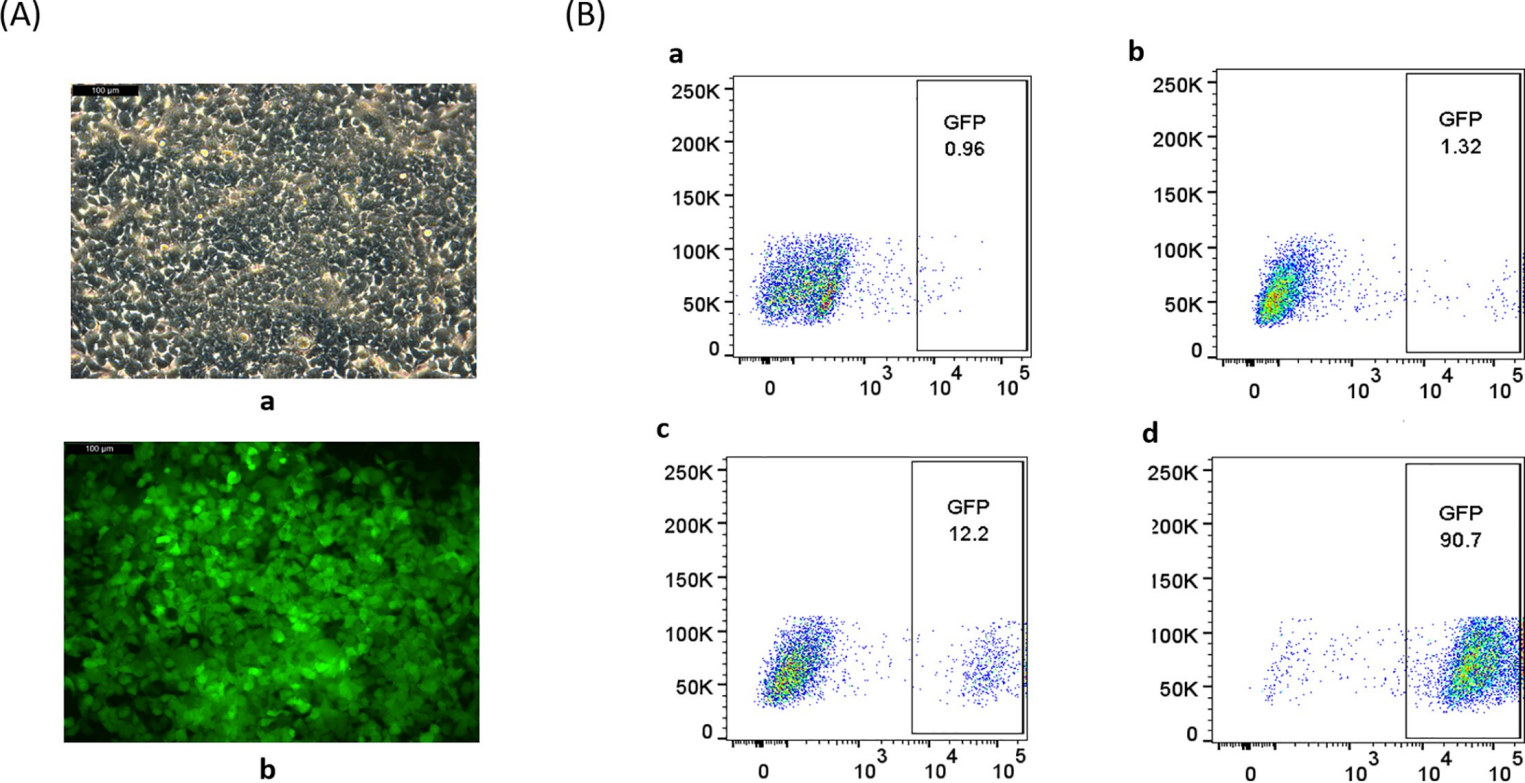
Evaluation of the efficiency of TARGATT site-specific integration in the HEK293T master cell line. (A) Fluorescence microscopy of the HEK293T master cell line transfected with the donor plasmid containing GFP and attB and treated with GCV. (a), bright-field; (b), GFP channel. (B) FACS analysis of GFP expression in HEK293T cells. (a), HEK293T parental cell; (b), HEK293T parental cell line transfected with the donor plasmid; (c), HEK293T master cell line transfected with the donor plasmid, prior to GCV selection; (d), HEK293T master cell line transfected with the donor plasmid, after GCV selection.

To examine whether the TARGATT-mediated integration system would be applicable to other cell types, we applied an analogous strategy to that used in CHO-S cells to the HEK293T cell line. First, a HEK293T master cell line was generated at the ROSA26 locus, using CRISPR/Cas9 homologous recombination technology. DNA sequencing results of the PCR products showed the landing pad had been inserted as expected into the ROSA26 locus (S6 Fig). Next, a donor plasmid containing GFP was knocked into the HEK293T master cell line via TARGATT recombination. After GCV selection, GFP expression was examined using fluorescence microscopy and FACS. Similar to our observations in the CHO-S cell system, fluorescence microscopy indicated a high percentage of cells expressing GFP after GCV selection (Fig 5A). FACS analysis showed 12.2% and 90.7% GFP-positive cells before and after GCV selection, respectively. In contrast, only 1.32% cells were GFP-positive after random insertion (Fig 5B). Similar to what we found in CHO-S cell system, western blotting showed an increase in GFP expression after GCV selection (S3 Fig, lanes 1–3), and ddPCR revealed that a single copy of GFP was present in the GCV-selected cell line (S5 Fig). DNA sequencing results of the PCR products showed the correct recombination sequences (S7 Fig).

## Discussion

We developed a strategy to achieve precise, rapid gene insertion in mammalian cells. We first established, using CRISPR/Cas9, master cell lines carrying a landing pad containing a pair of PhiC31 attP sites. Then, integrase based TARGATT technology was used to knock-in a GOI. We demonstrated that post-selection gene integration efficiency as high as 97.7% can be achieved with this process. We validated the system in two cell lines at two unrelated loci, suggesting that it is applicable to different mammalian cell lines, targeting various genomic loci.

In our strategy, the master cell line landing pad construct contains two attP sites from bacteriophage PhiC31. Between the two attP sites is a cassette that co-expresses three proteins. The first protein enables puromycin selection, which is used to enrich for cells with on-target landing pad insertion. It can be replaced with any other positive-selection marker, such as the glutamine synthetase gene. HSV-TK, the second expressed protein, is included to enable selection of on-target knock-in of the GOI. An on-target knock-in would lead to replacement of the landing pad cassette with the GOI. Through GCV treatment, parental cells and those with random insertion, all containing the HSV-TK protein, would be eliminated, leaving only the cells with the correct insertion. The third protein, PhiC31 integrase, makes TARGATT recombination possible. Once the donor construct, containing two attB sites and the transgene, is introduced into the cells, PhiC31 integrase mediates integration into the landing-pad locus. Successful integration will replace all three proteins in the landing pad, so that the product after integration has no other newly introduced genes except the GOI. We included an HSV-TK gene in the donor construct backbone, so all cells with the plasmid backbone integrated into the genome would also be eliminated by GCV selection. The final cells selected, therefore, are unlikely to contain the plasmid backbone.

This system has several advantages over traditional procedures for generating cell lines for protein production. The first is high efficiency. Previous research showed that site-specific recombination in CHO-S cells using other techniques was relatively low. In CHO-S cells, the efficiency of non-homologous end joining (NHEJ) targeted integration was only 0.45% and it was 7.4%–27.8% using the CRISPR/Cas9 system after selection with G418, 6-TG or m-Cherry [22, 23]. Our system reached an efficiency of 97.7% after selection by GCV. Furthermore, as the size of the DNA fragment is increased, the efficiency of insertion through random or CRISPR-based integration is dramatically decreased [24]. In contrast, integrase-based knock-in has virtually no size limit [25]. Thus, the efficiency advantage of our system will likely become even more apparent with larger insertion sizes. It could also be more advantageous for expression of more than one gene in the same cell. The second advantage of our system is the short turnaround time. Instead of the several weeks that are required for single cell cloning, our system only requires a few days to perform GCV counter-selection to obtain the final stable cell line. Once a validated master cell line is generated with a landing pad inside, it will require only two to three weeks to generate a stable cell line expressing the GOI. A third advantage is consistency and homogeneity. By introducing HSV-TK negative selectable markers to eliminate cells with unwanted recombination events after cassette exchange, homogeneity of the cell population in the final line is ensured. Because this system employs single-site insertion, there is no gene amplification required, but the methodology would also be compatible with gene amplification. For single copy or other low copy-number insertions, there would be a lower chance of gene rearrangements, so the final cell lines will be much more stable than those obtained by random integration. Furthermore, the final cells will have no plasmid backbone, avoiding negative side-effects like gene silencing that could lead to decreased protein expression. Overall, with this system, the final cell populations obtained will be more homogenous at both the genomic and gene expression levels.

One of the critical elements for successful use of our system is a locus in the genome with high gene expression. In CHO-S cells, we confirmed that the orthologous Hipp11 locus meets this expression requirement. Using our system, we generated a cell line expressing GFP at high levels. The GFP-positive signal strength did not decrease in cells cultured for over four months, supporting the promise of Hipp11 as a "safe harbor" genomic locus for stable, efficient transgene knock-in and expression. Finally, based on its successful implementation in HEK293T cells, we propose that our strategy is applicable to many loci in various mammalian genomes.

## Supporting information

S1 Fig Genotyping of the CHO-S master cell line by Sanger sequencing. Sequence alignments of the Sanger sequence of the PCR products and the genomic locus of H11 with on-target CRISPR knock-in of the landing pad. (A). Alignment of the 5’arm (GenBank accession number MN167150). (B). Alignment of the 3’arm (GenBank accession number MN167151).
(DOCX)

S2 Fig Evaluation of the off-target sites. (A) Alignments of the top 5 potential sites and Cas-gRNA2. The mismatched bases were highlighted in red. (B) PCR amplification of the top 5 potential sites by designed primers. (C) Sequence alignment of CHO-S genome sites 1–5 and Sanger sequence results (GenBank accession number MN167154, MN167155, MN167156, MN167157 and MN16758). Primer used for amplifying the sites were highlighted in purple. Sites 1–5 were highlighted in blue.
(DOCX)

S3 Fig GFP protein expression analyzed by Western blotting. (1), HEK293T master cell line only; (2), HEK293T master cell line transfected with the donor plasmid by TARGATT, prior to GCV selection; (3), HEK293T master cell line transfected with the donor plasmid by TARGATT, after GCV selection. (4), CHO-S master cell line only ("4–6"); (5), CHO-S master cell line ("4–6") transfected with the donor plasmid, prior to GCV selection; (6), CHO-S master cell line ("4–6") transfected with the donor plasmid, after GCV selection.
(DOCX)

S4 Fig On-target knock-in and correct recombination between the attP and attB docking sites in the CHO-S cell line. (A). Sequences alignment of 5’ recombination site (GenBank accession number MN167152). (B). Sequences alignment of 3’ recombination site (GenBank accession number MN167153). The resultant recombination sites (attL and attR) between attP and attB were labeled in magenta.
(DOCX)

S5 Fig GFP copy numbers were analyzed by ddPCR. (A) The values of GFP copy numbers from CHO-S and HEK293T master cells (transfected with donor plasmid, after GCV selection) were shown in table in two repeats. (B) GFP copy numbers from CHO-S and HEK293T cells (transfected with donor plasmid, after GCV selection) were shown in columns.
(DOCX)

S6 Fig Genotyping of the HEK293T master cell line by Sanger sequencing. Sequence alignments of the Sanger sequence of the PCR products and the genomic locus of ROSA26 with on-target CRISPR knock-in of the landing pad. (A). Alignment of the 5’arm (GenBank accession number MN167159). (B). Alignment of the 3’arm (GenBank accession number MN167160).
(DOCX)

S7 Fig On-target knock-in and correct recombination between the attP and attB docking sites in the HEK293T cell line. (A). Sequences alignment of 5’ recombination site (GenBank accession number MN167161). (B). Sequences alignment of 3’ recombination site (GenBank accession number MN167162). The resultant recombination sites (attL and attR) between attP and attB were highlighted in magenta.
(DOCX)
