# Supplementary figures and images for: A system for site-specific integration of transgenes in mammalian cells
Source: PLoS One. 2019 Jul 25;14(7):e0219842. doi: 10.1371/journal.pone.0219842 (PMC6657834; doi:10.1371/journal.pone.0219842)

(A)


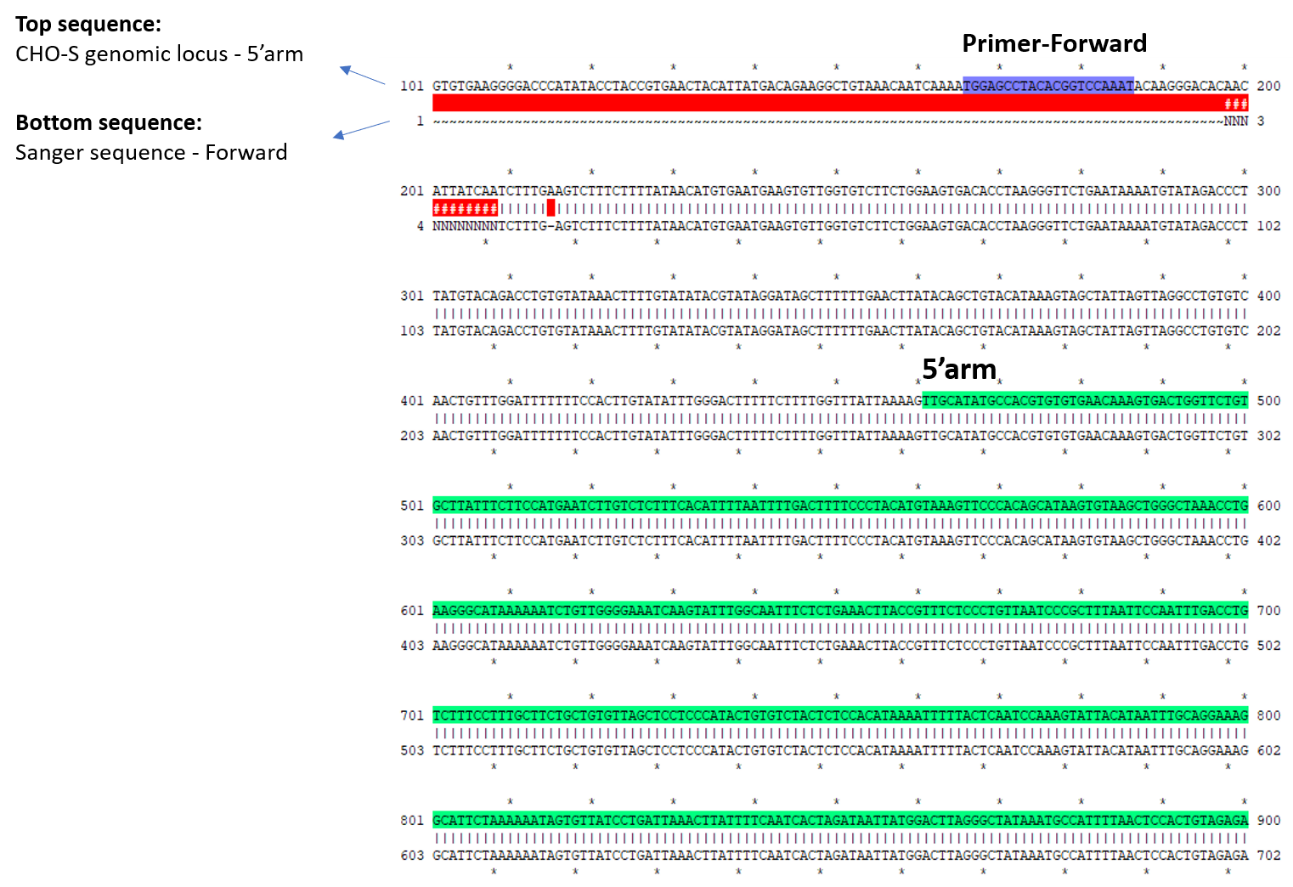


(B)


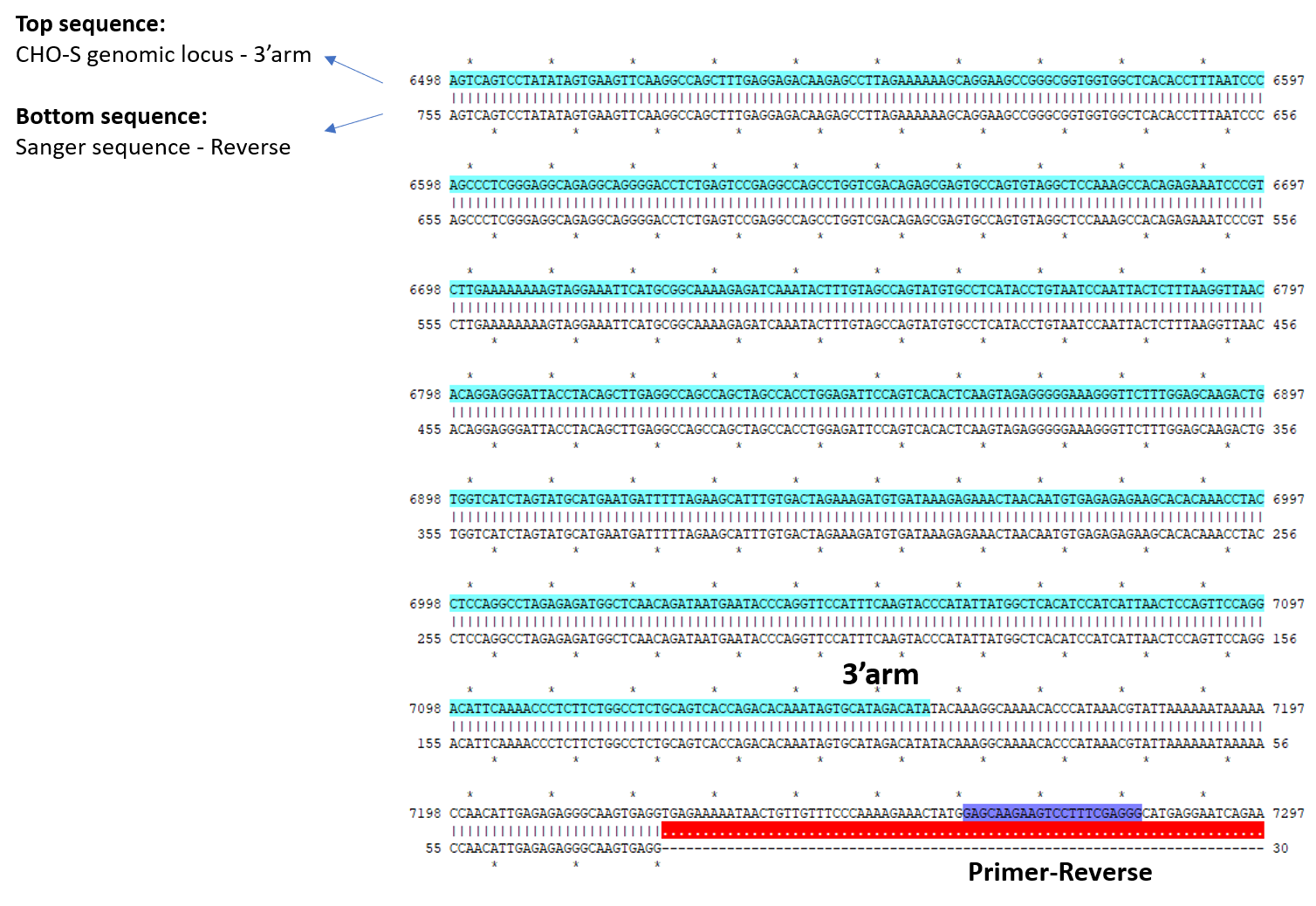

Supplement: S1 Fig — Sequence alignments of the Sanger sequence of the PCR products and the genomic locus of H11 with on-target CRISPR knock-in of the landing pad. (A). Alignment of the 5’arm (GenBank accession number MN167150). (B). Alignment of the 3’arm (GenBank accession number MN167151). (DOCX) [file pone.0219842.s001.docx]

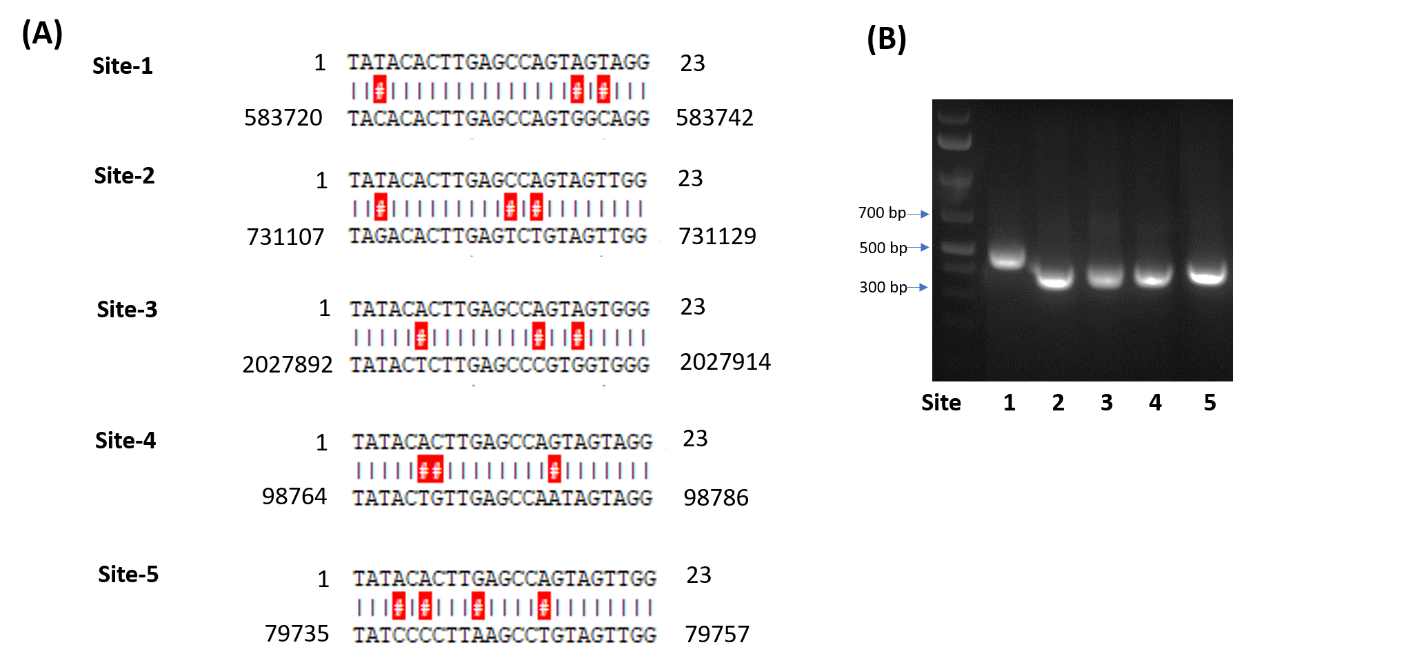


(C)
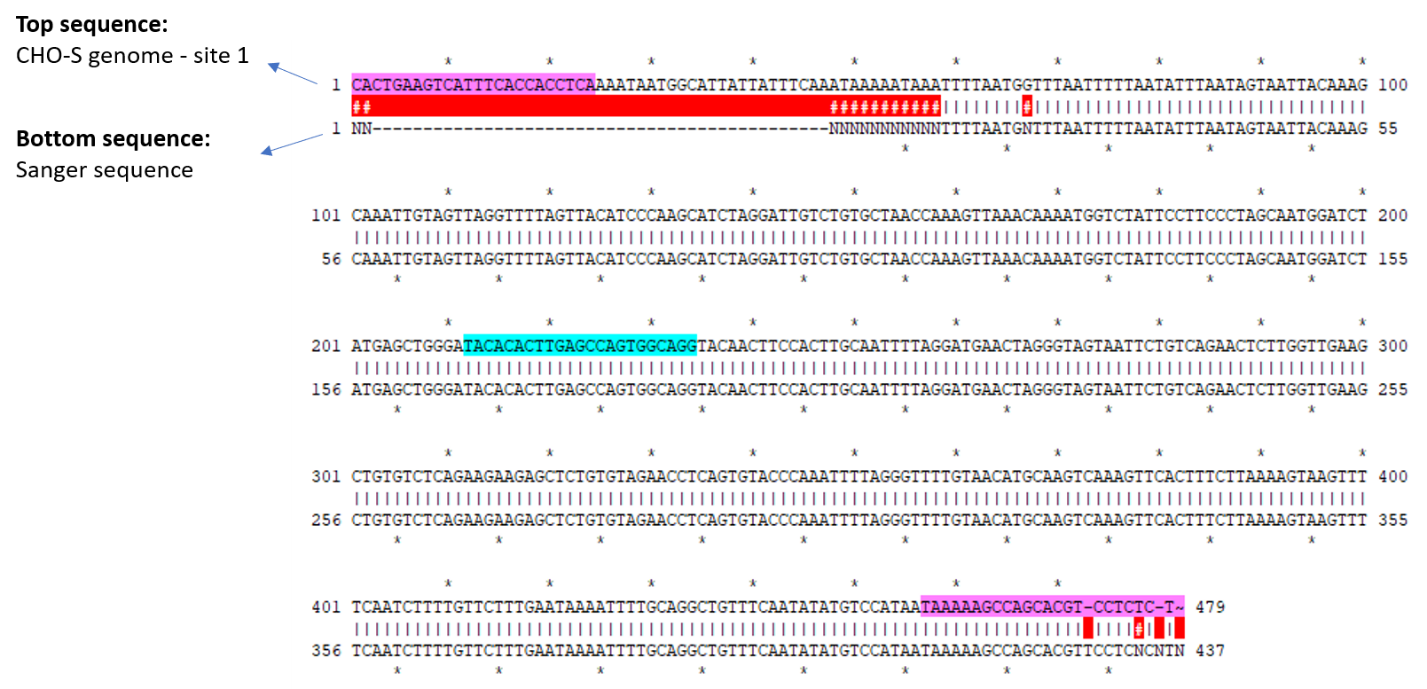


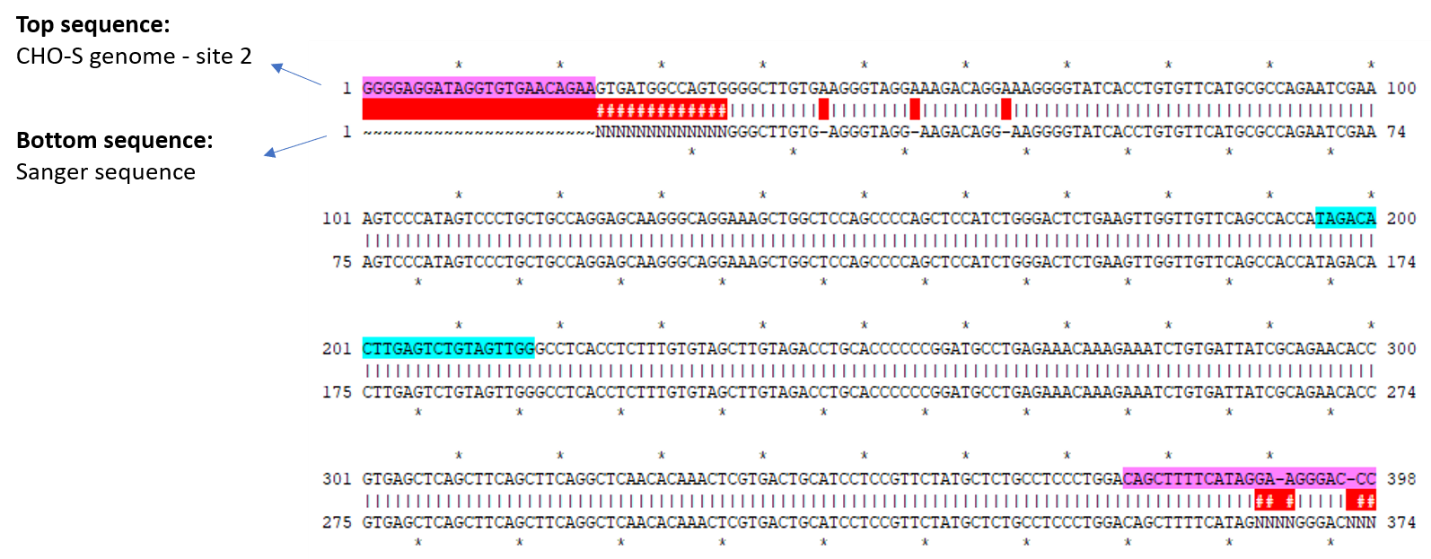


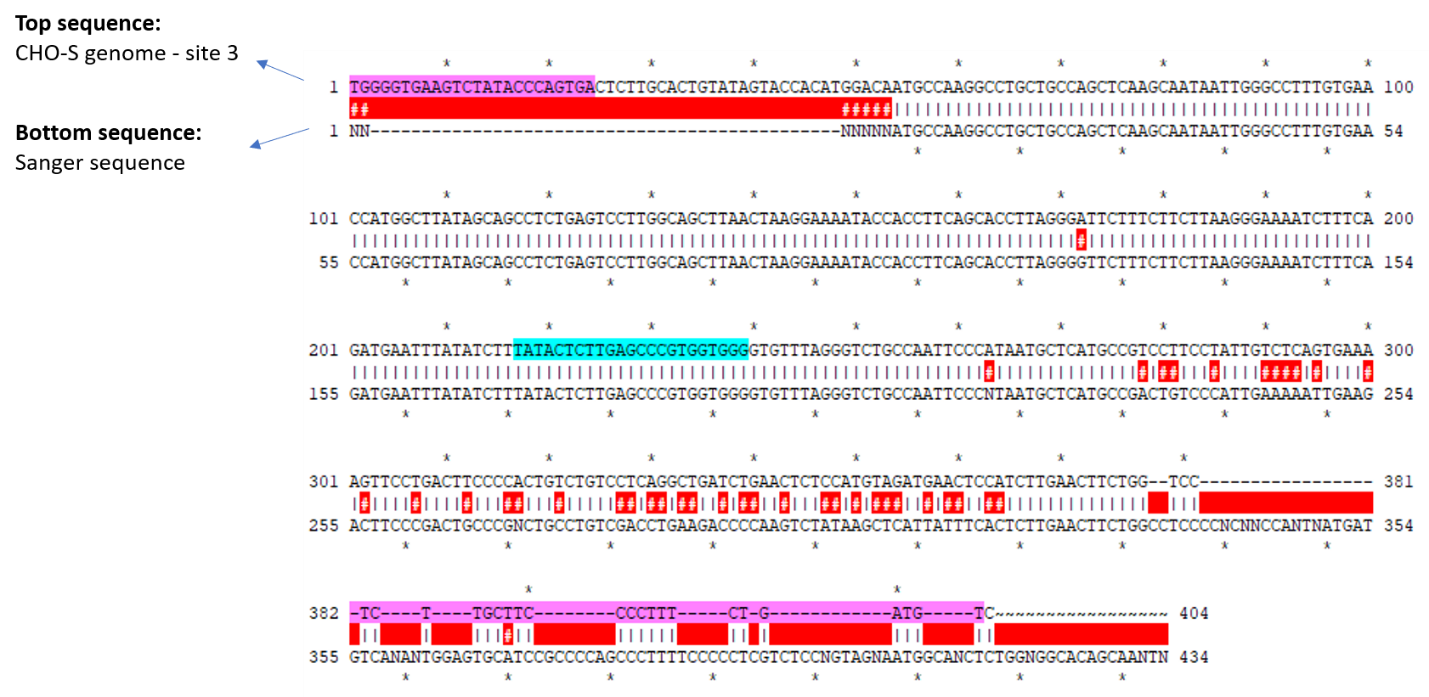


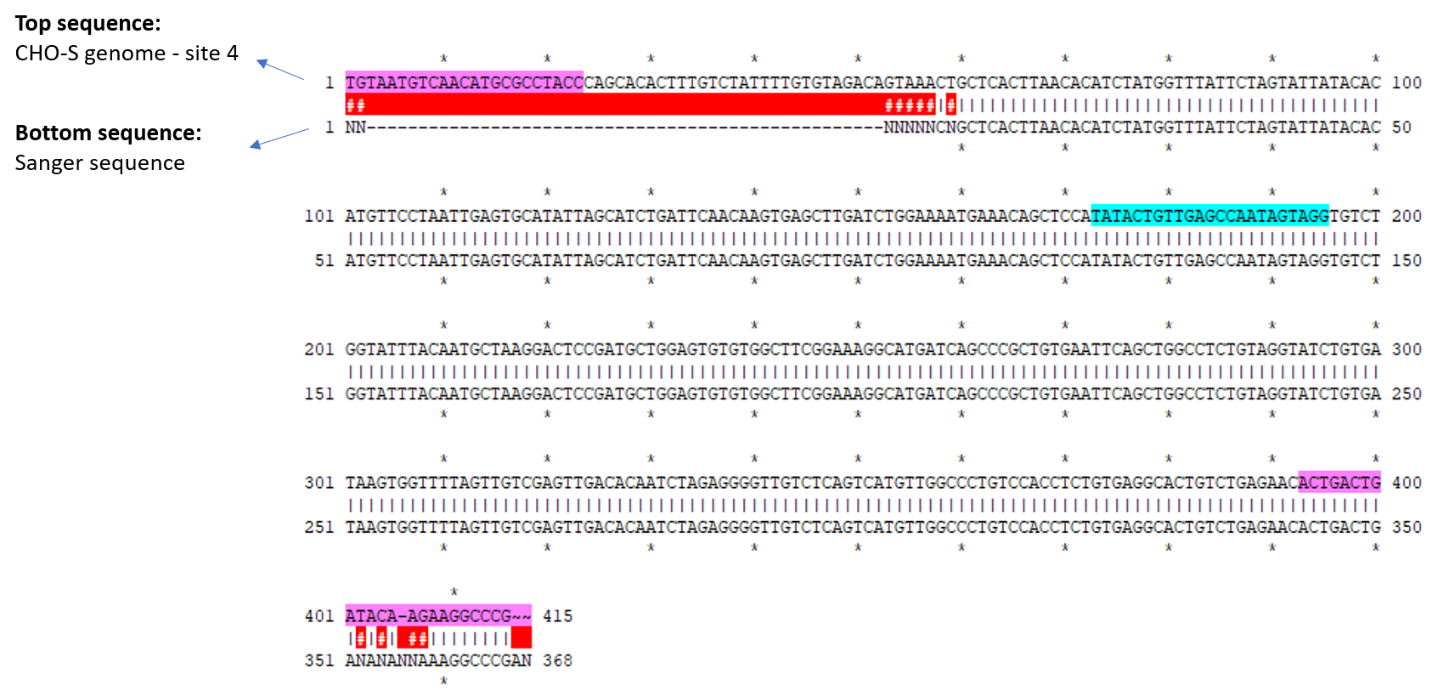


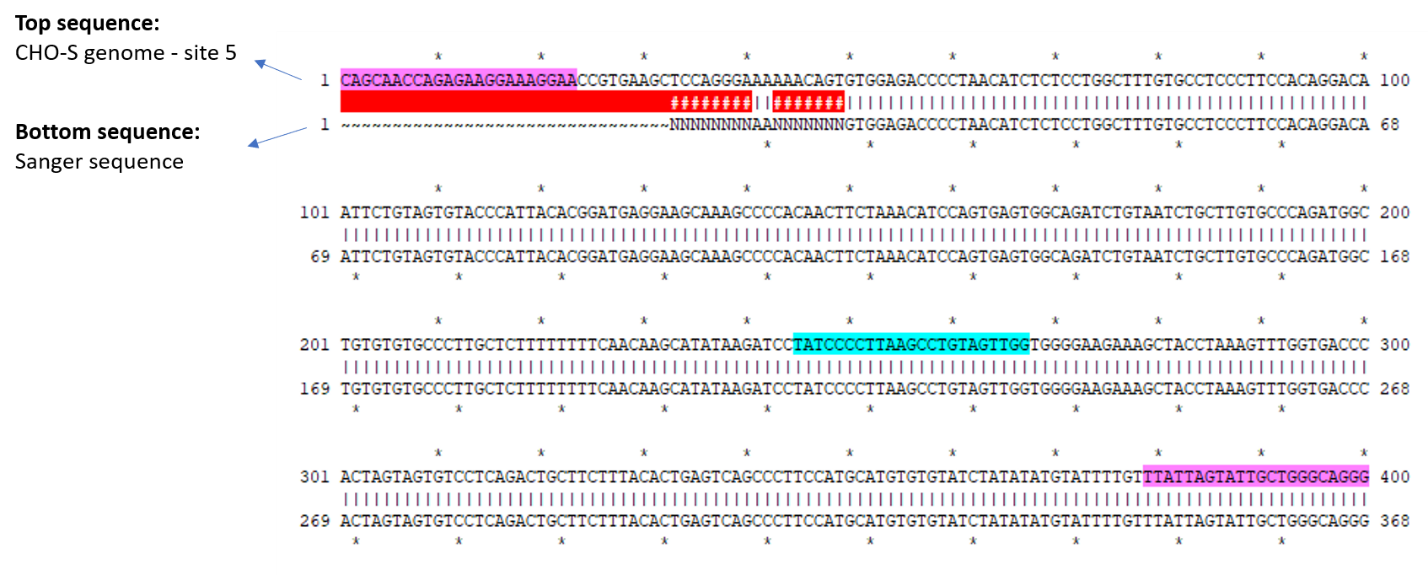

Supplement: S2 Fig — (A) Alignments of the top 5 potential sites and Cas-gRNA2. The mismatched bases were highlighted in red. (B) PCR amplification of the top 5 potential sites by designed primers. (C) Sequence alignment of CHO-S genome sites 1–5 and Sanger sequence results (GenBank accession number MN167154, MN167155, MN167156, MN167157 and MN16758). Primer used for amplifying the sites were highlighted in purple. Sites 1–5 were highlighted in blue. (DOCX) [file pone.0219842.s002.docx]

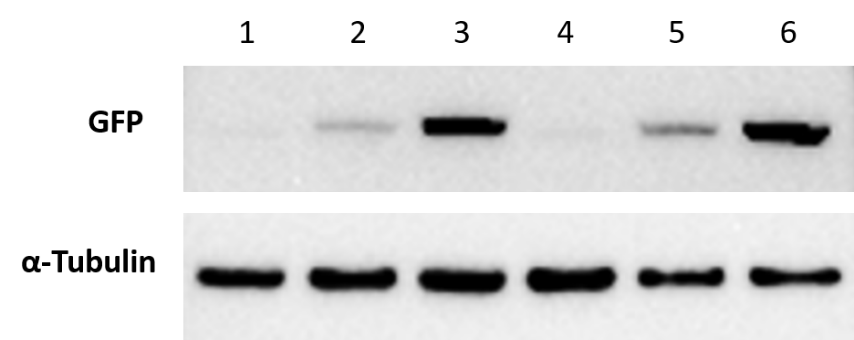

Supplement: S3 Fig — (1), HEK293T master cell line only; (2), HEK293T master cell line transfected with the donor plasmid by TARGATT, prior to GCV selection; (3), HEK293T master cell line transfected with the donor plasmid by TARGATT, after GCV selection. (4), CHO-S master cell line only ("4–6"); (5), CHO-S master cell line ("4–6") transfected with the donor plasmid, prior to GCV selection; (6), CHO-S master cell line ("4–6") transfected with the donor plasmid, after GCV selection. (DOCX) [file pone.0219842.s003.docx]

(A)
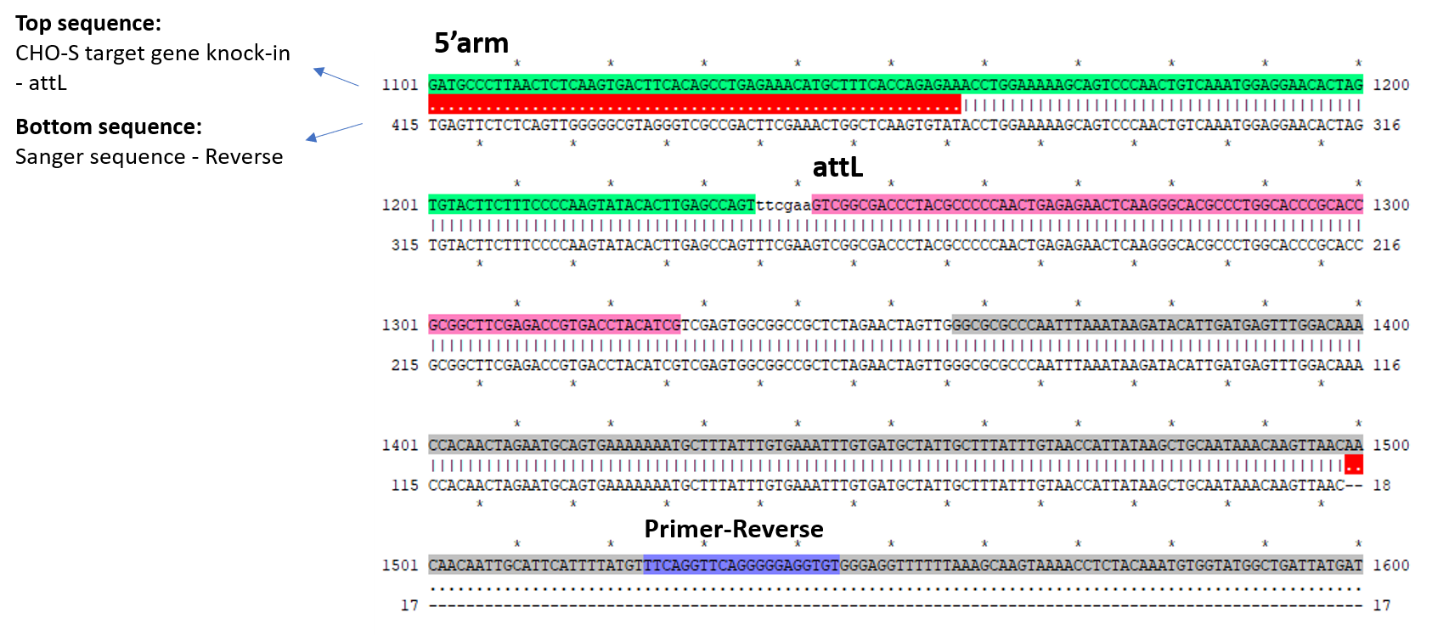


(B)
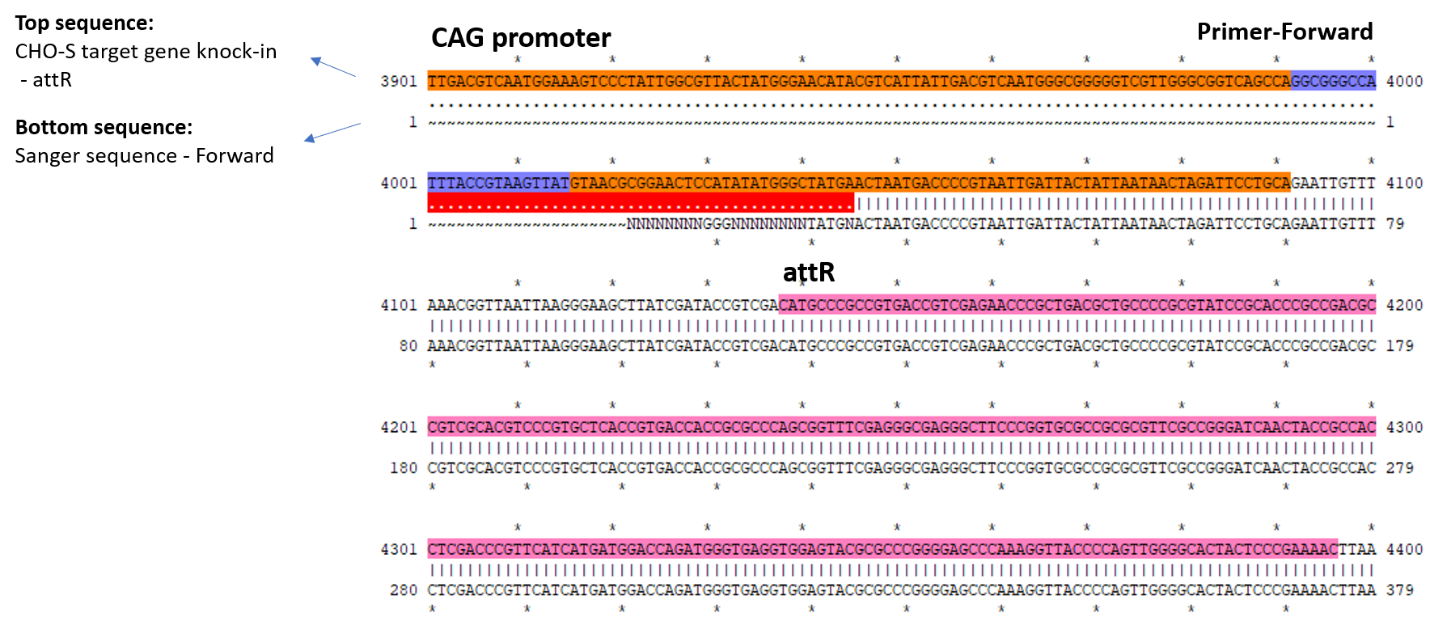

Supplement: S4 Fig — (A). Sequences alignment of 5’ recombination site (GenBank accession number MN167152). (B). Sequences alignment of 3’ recombination site (GenBank accession number MN167153). The resultant recombination sites (attL and attR) between attP and attB were labeled in magenta. (DOCX) [file pone.0219842.s004.docx]

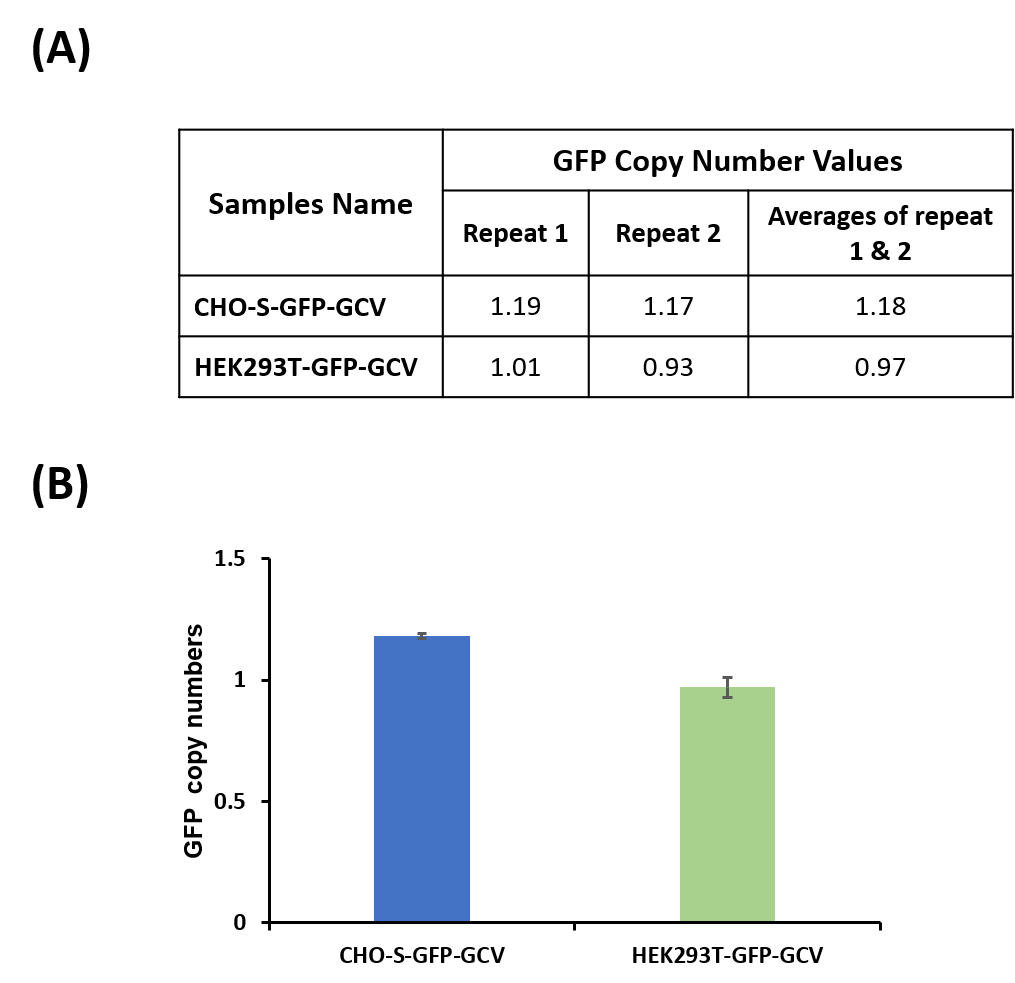

Supplement: S5 Fig — (A) The values of GFP copy numbers from CHO-S and HEK293T master cells (transfected with donor plasmid, after GCV selection) were shown in table in two repeats. (B) GFP copy numbers from CHO-S and HEK293T cells (transfected with donor plasmid, after GCV selection) were shown in columns. (DOCX) [file pone.0219842.s005.docx]

(A)
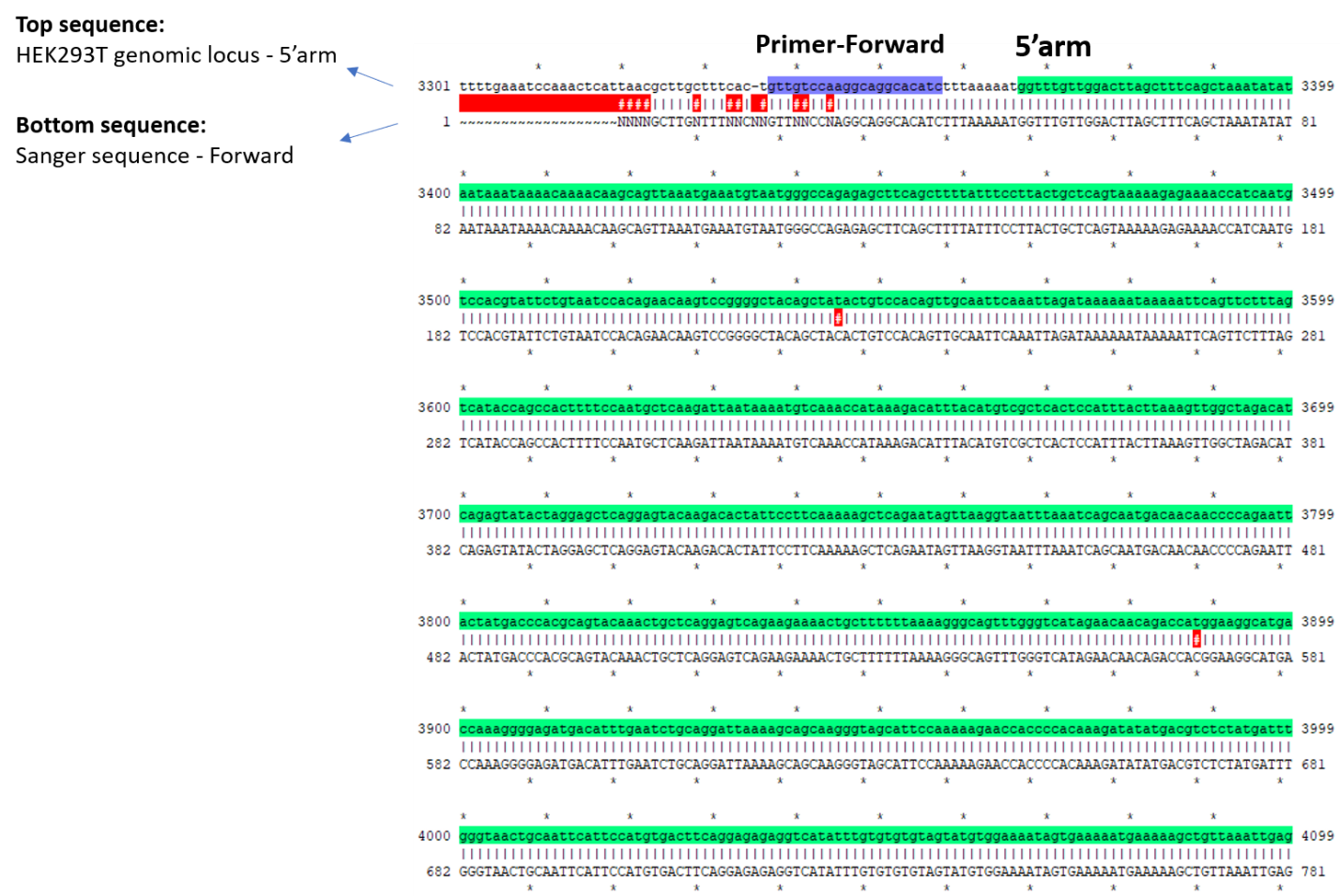


(B)
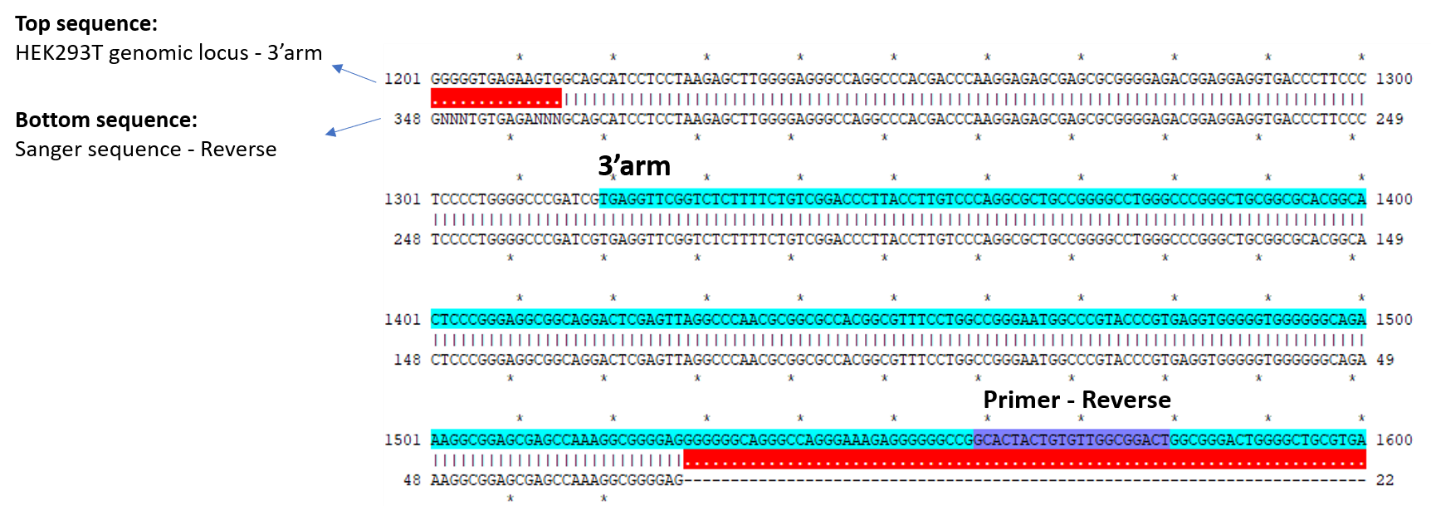

Supplement: S6 Fig — Sequence alignments of the Sanger sequence of the PCR products and the genomic locus of ROSA26 with on-target CRISPR knock-in of the landing pad. (A). Alignment of the 5’arm (GenBank accession number MN167159). (B). Alignment of the 3’arm (GenBank accession number MN167160). (DOCX) [file pone.0219842.s006.docx]

(A)
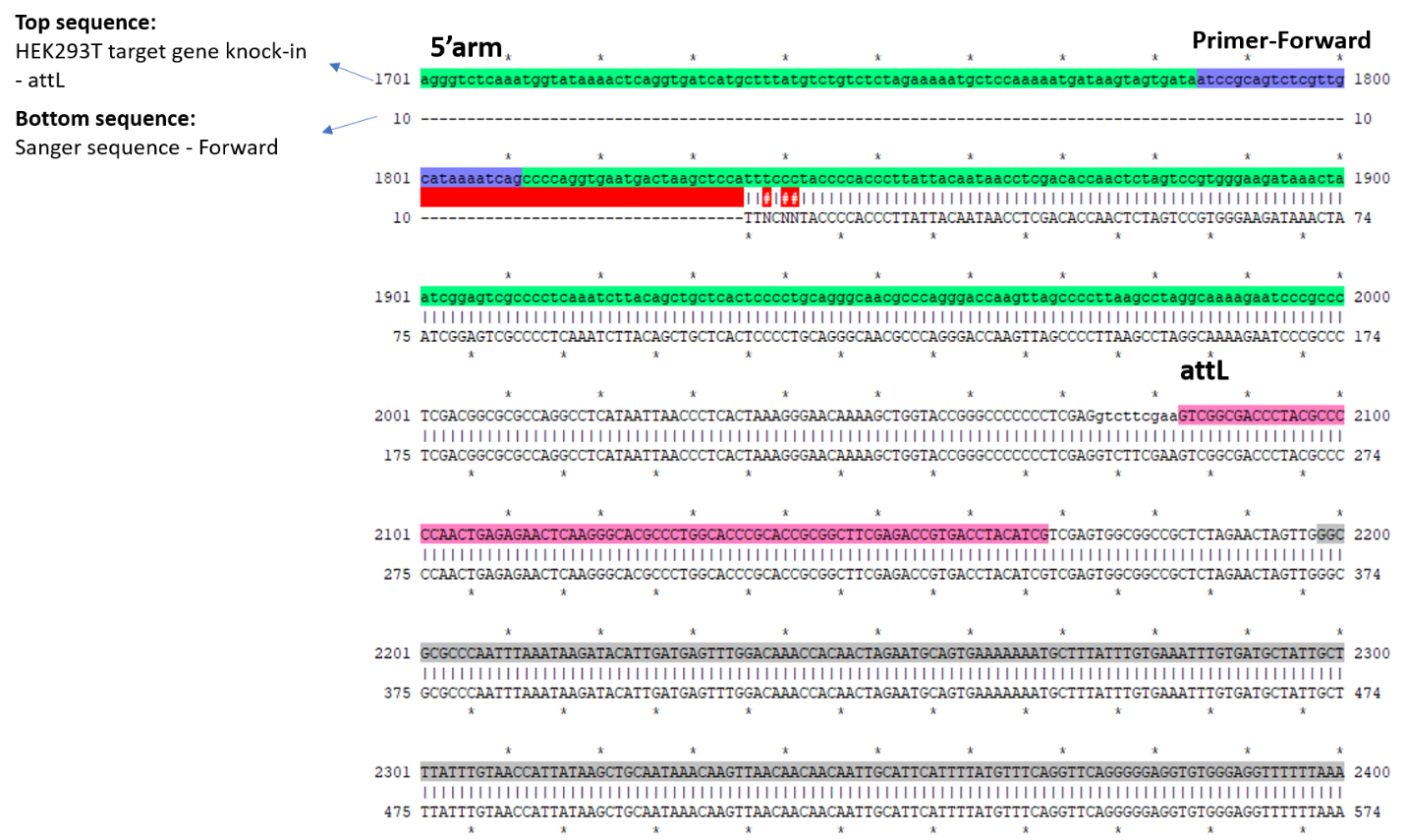


(B)
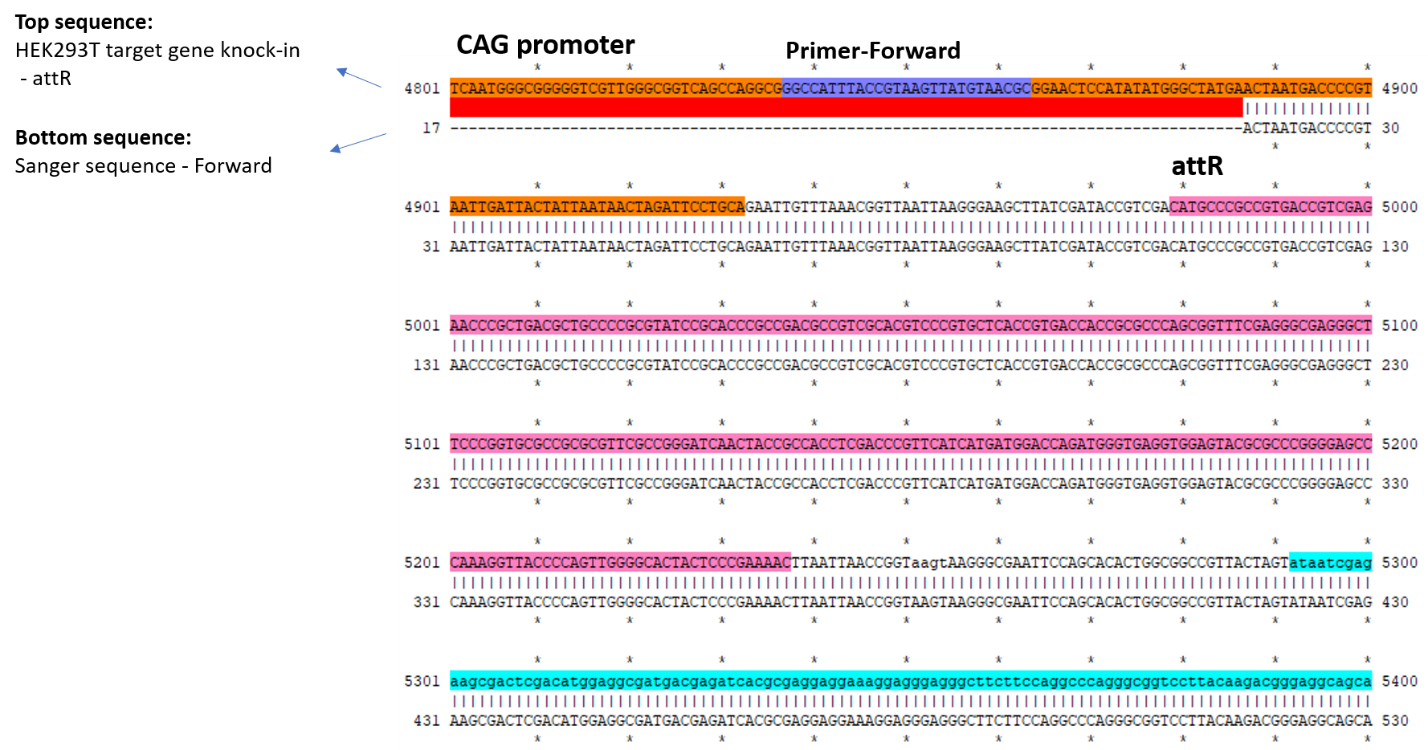

Supplement: S7 Fig — (A). Sequences alignment of 5’ recombination site (GenBank accession number MN167161). (B). Sequences alignment of 3’ recombination site (GenBank accession number MN167162). The resultant recombination sites (attL and attR) between attP and attB were highlighted in magenta. (DOCX) [file pone.0219842.s007.docx]
